# Intervention in autism based on Early Start Denver Model in a multiethnic immigrant setting—experiences of preschool staff involved in its implementation

**DOI:** 10.3389/frcha.2024.1341729

**Published:** 2024-03-13

**Authors:** Petra Linnsand, Gudrun Nygren, Jonas Hermansson, Christopher Gillberg, Emilia Carlsson

**Affiliations:** ^1^ Gillberg Neuropsychiatry Centre, Institute of Neuroscience and Physiology, University of Gothenburg, Gothenburg, Sweden; ^2^ Child and Adolescent Specialist Centre, Angered Hospital, SV Hospital Group, Gothenburg, Sweden; ^3^ Research Department, Angered Hospital, SV Hospital Group, Gothenburg, Sweden; ^4^ Speech and Language Pathology Unit, Institute of Neuroscience and Physiology, University of Gothenburg, Gothenburg, Sweden

**Keywords:** autism, early start denver model, preschool setting, multiethnic immigrant setting, qualitative study

## Abstract

**Background:**

Early interventions for young children with autism have been shown to enhance developmental outcomes. However, opportunities for targeted interventions in autism, both in care and preschool, are often lacking, particularly in immigrant communities. The early start denver model (ESDM) stands as one of the most well-established intervention models, including improvement in core developmental domains and reduction of maladaptive behaviours, also delivered in preschool settings. An intervention program based on the ESDM was implemented in collaboration with parents, preschool staff, and health care professionals locally in a multiethnic immigrant and socioeconomically disadvantaged area in Gothenburg, Sweden.

**Purpose:**

The present study aimed to describe a low intensity intervention program based on the ESDM for young children with autism in a multiethnic immigrant setting and capture the experiences of the preschool staff involved in implementing the program.

**Method:**

Fifteen preschool professionals were interviewed through focus group interviews. Data were analyzed using content analysis. The interviews focused on capturing the crucial factors in the intervention program and to get more in-depth information about the intervention program's influence on the children with autism, their parents, the preschool staff, and preschool activities.

**Results:**

Central components of the intervention program were emphasized by the preschool staff. These encompassed contextual prerequisites, such as the preschool staff's participation in intervention program fostered by the local environment and features linked to the ESDM methodology. The preschool staff emphasized that shared objectives and regular network meetings as fundamental components of the model. They also underscored the seamless alignment between the ESDM and the preschool curriculum emphasizing how ESDM strategies could be easily integrated into the preschool's daily routines. The staff's experience indicated that the implementation of ESDM enriched the learning experiences of children with autism and yielded benefits to their parents, fellow peers in the preschool, and the preschool staff.

**Conclusions:**

The intervention program based on the ESDM presents a promising model for young children with autism in a multiethnic immigrant setting. Several critical factors based on the preschool staff's experiences were essential for implementation: contextual prerequisites such as organizational support, close collaboration with healthcare professionals, good competence among preschool staff, and the ESDM itself.

## 1 Introduction

A review study from 2022 estimates that approximately 1/100 children worldwide receive an autism diagnosis, yet the figures diverge considerably (1). With a prevalence of 2.15% in children aged four years (2), autism accounts for one of the most substantial costs among mental disorders afflicting children globally (3). Several researchers have highlighted the importance of early identification of autism signs (4–6), as the first years of life have been underscored as a critical period characterized by significant neural plasticity affording a greater potential to influence the developmental course of brain growth (7, 8).

Early interventions are essential, both for treating prodromal symptoms and manifested autism (9, 10). Zwaigenbaum et al. (9) have summarized recommendations for early interventions, (a) interventions for children aged <3 years with suspected or confirmed autism should integrate both developmental and behavioural approaches and begin as early as possible, (b) active involvement of families and/or caregivers in the intervention process, (c) comprehensive intervention addressing both core symptoms and related difficulties, and (d) the sociocultural beliefs and socioeconomic aspects of the family should be considered in the intervention services.

A diverse array of early intervention programs caters to young children with autism. Some intervention programs focus on a specific developmental domain and other programs are more comprehensive (11–13). Also, there are numerous ways in which and by whom the interventions can be delivered. Naturalistic Developmental Behavioural Interventions (NDBIs) constitute a group of interventions that use strategies from applied behavior analysis (ABA) but also strategies retrieved from developmental science to teach developmentally appropriate skills. The NDBIs have moved the focus from isolated teaching episodes towards teaching in natural settings based on the child's interest and motivation (14). In a review, Crank et al. (15) found that NDBIs may increase language, social communication, play skills, and cognition in young children with autism.

The early start denver model (ESDM) is one of the most well-established NDBIs and has been widely studied (16). The ESDM has been evaluated in several studies, systematic reviews, and meta-analyses (17–19). The reviews concluded that the ESDM is a promising intervention for young children with autism, with positive outcomes in, for example, cognitive and language domains. Also, from a low resource setting in South Africa, ESDM has been reported to be promising, but more research is necessary (20).

The ESDM is a comprehensive intervention, designed for children aged 12–48 months. Based on behavioural science, developmental psychology, and neuroscientific evidence, the ESDM aims to reduce the severity of autism symptom and improve the development of cognitive, social, emotional, and language abilities (17, 18, 21, 22). To build a strong learning structure, the ESDM focuses on developing a wide range of skills, for example imitation, shared affect, joint attention, turn-taking, and attending to others, then those skills have been identified as predictors of later communication, language, and cognition. A manualised curriculum, dived into four steps, is used to create objectives within nine developmental areas, including receptive and expressive communication, social skills, imitation, gross and fine motor skills, and adaptive functioning skills. The learning context is the child's typical daily activities, routines, and interactions in which the adult creates opportunities for learning and interaction. In the ESDM, the child's interest and motivation are essential, and will lead the adult in interaction with the child (16).

Parents have a crucial role in early interventions provided to young children with autism (9). Parent-implemented ESDM (P-ESDM), in which parents are coached to deliver the intervention, maximizes learning opportunities in daily activities and bridge service gaps (23). Several studies on the ESDM have focused on evaluating the effects of parent delivered intervention. The studies have demonstrated that P-ESDM is effective for improving parent use of ESDM strategies, reducing parenting stress, and improving child outcomes in different domains (12, 21, 24–26).

Further, the ESDM has been delivered within group day care settings, both in inclusive and specialized environments, i.e., autism specific preschools (27–30). Vivanti et al. (31) have studied different factors associated with social communicative outcomes for children receiving Group-based ESDM (G-ESDM) in inclusive vs. autism-specific classrooms. They found that G-ESDM improved the child's spontaneous vocalisation, social interaction, spontaneous imitation, verbal cognition, parent-reported autism symptom presentation, and adaptive functioning, irrespective of the interventions setting. However, children with higher social interest and nonverbal cognitive skills might benefit more from the inclusive educational setting. Further, Sinai-Gavrilov et al. (32) have shown that, compared to a multidisciplinary developmental intervention group, children in a preschool based ESDM group made more significant gains in overall cognitive development, language skills, adaptive communication, and socialisation abilities. In addition to improvements in core development domains, Fulton et al. (30) have demonstrated that preschool based ESDM may effectively decrease maladaptive behaviour in children with autism.

Few studies have reported how preschool staff experience working with the ESDM in preschool settings. Tupou et al. (33) have explored preschool teachers' experiences of an ESDM coaching program in inclusive preschool settings. The teachers perceived the program to be both highly acceptable and effective. For example, they experienced that the ESDM strategies fit naturally into their usual teaching practice and that the intervention positively impacted their own knowledge and confidence as well as the children's communication, engagement, and relationships. Also, Holzinger et al. (34) found that preschool teachers regarded the ESDM as practicable in regular preschool setting, such as the objectives and principles could be integrated into group routines.

In Sweden, Early Intensive Behavior Therapy (EIBI) is the most common intervention model for young children with autism. Earlier studies focusing on EIBI in preschool settings reported challenges when working with the EIBI in regular preschools (35). In a study by Kvick (36), the preschool staff described obstacles in collaboration with healthcare services, including different perspectives on learning, challenges in aligning goals within the preschool activities, and transfer of the child's skills from training situations to daily activities.

Preschool education is the initial stage in the Swedish educational system, and it is included in the Swedish Education Act (37). The Swedish preschool curriculum encapsulates the preschool's fundamental values, goals, and guidelines (38). In Sweden, the majority of children with special educational needs attend regular preschools. The Swedish Education Act emphasizes equality, with the onus on preschools to support children with special needs ensuring their optimal development (37). Preschool staff in Sweden mainly include preschool teachers (university degree in Preschool Education) and preschool care workers (upper secondary education) (39).

In Sweden, 26.9% of the total population has have non-Swedish backgrounds^1^, but in some districts the proportion of immigrants is considerably higher (40). Several studies have found an increased prevalence of autism among immigrants and multiethnic minorities (2, 41–43). In addition, immigrant parents have reduced access to health care and regular support sources compared to nonimmigrant parents (44, 45). For example, studies have shown that after receiving an autism diagnosis, children of immigrants are less likely to access health care services, such as interventions (46–48). To provide accessible assessment and interventions for preschool children with autism, a multidisciplinary team was established in one multiethnic and socioeconomically disadvantaged district of Gothenburg, Sweden. For more information about the team and intervention program, see Methods.

The multidisciplinary team developed a low-intensity program based on the ESDM for children with autism from a multiethnic immigrant background. The aim of the present study was to (1) describe the low-intensity intervention program based on the ESDM in a multiethnic immigrant setting and (2) capture the experiences and perspectives of the preschool staff involved in implementing the intervention program.

Specifically, we addressed the following questions:

1. What do the preschool staff experience as key elements in the intervention program based on ESDM that promote optimal development for young children with autism?
2. What do the preschool staff experience as facilitators and barriers in general for providing the intervention program to young children with autism?
3. How do the preschool staff experience that the intervention program influences the children with autism, their parents, the preschool staff as well as the preschool activities?

## 2 Methods

### 2.1 Study design

A qualitative explorative study with semi-structured focus group interviews was conducted (49, 50). We attempted to acquire a better understanding of how a preschool intervention program for autism based on the ESDM was experienced by those delivering it, i.e., the preschool staff. Qualitative research is beneficial when researching topics involving participants' in-depth experiences (51). Unlike individual interviews, focus groups offer an added dimension of member interactions (52). The group members are facilitated to communicate, exchange anecdotes and comment on each other's experiences or points of view. In this way, focus groups can capture dimensions of understanding that often remain untapped or unreachable by other data collection forms (52–54). The interviews were analysed through qualitative content analysis, a method that can be used to analyse the content in communication, such as interviews, systematically (55, 56).

### 2.2 Study context

The study was conducted in a multiethnic and socioeconomically disadvantaged area in Gothenburg, Sweden, with ∼10,080 inhabitants, of whom ∼770 were aged 1–5 years. In this area, 91% of the population have a non-Swedish background, compared to the 39.1% citywide average in Gothenburg. The most common birth countries are Somalia (10.2%), Iraq (8.6%), and Syria (7.2%). Ill health, high unemployment rate, and low average income is prevalent in the area (57). In an earlier study from the area, we reported a high prevalence of autism, 3.7%, in preschool children. For more information about the study area and child characteristics see Linnsand et al. (58).

To meet the families' extensive needs, as well as to handle the linguistic, cultural, and socioeconomic barriers, a multidisciplinary team was established in the study area. In collaboration with preschools and other social services, the multidisciplinary team provided assessment and interventions for families with children aged 1–4 years affected by neurodevelopmental difficulties, particularly autism. The program's primary objectives were to facilitate assessment and intervention to these families locally, thereby enhancing the accessibility and continuity of health care services, while also encouraging active parental participation in the interventions. For example, the clinic was located centrally in the area to increase accessibility to health care. An individual treatment plan was designed based on the child's needs and the entire family's needs and prerequisites. For more information about the multidisciplinary team, see Nilses et al. (59), Linnsand et al. (58), and Nygren et al. (60).

The intervention program for the children with autism was a low-intensity program based on the ESDM (referred to as the intervention program based on ESDM or intervention program) (16, 23). For more information about the intervention program, see 2.10 Procedure.

In Sweden, preschool is not compulsory, but most children attend preschool from 1 to 2 years of age. Among two-year-olds, 91.3% attend preschool, compared to 82.7% of those hailing from non-Swedish backgrounds^2^ (39). In the study area, there were 10 preschools. Each section had an average of 15.3 children, and each fulltime employee had an average of 4.8 children. Of the preschool staff, 39.3% were preschool teachers (university degree in Preschool Education), and 55.3% were preschool care workers (upper secondary education) (unpublish statistics from the city of Gothenburg).

### 2.3 Participants

Fifteen staff members were recruited from six preschools in the studied area. Their total experience implementing the intervention program based on the ESDM ranged from 3 to 7 years (mean 5.2, SD 1.3). Six of the participants were preschool care workers, five preschool teachers, three principals, and one was special education teacher. All participants were female. Their collective experience in the preschool setting ranged from 4 to 42 years (mean 20.3, SD 12.2). All participants had worked with at least two children in the intervention program (range 2–25).

The participants were divided into three focus groups; one group consisted of principals and a special education teacher (four participants), and two groups with preschool teachers and preschool care workers (five vs. six participants in each group) (Table 1).

**Table 1:** Participant demographics (*n* = 15).

Focus group (no)	Professional title	Sex	Professional years in preschool	Number of children with autism that the participant had worked with in the program
	Principal^a^	F	40	8–10
1	Principal^a^	F	25	15–25
	Principal^a^	F	35	15–20
	Special education teacher^b^	F	32	10–15
	Preschool teacher^c^	F	42	3
2	Preschool teacher^c^	F	17	3
	Preschool teacher^c^	F	16	3
	Preschool teacher^c^	F	9–10	2
	Preschool care worker^d^	F	19	“Several”
	Preschool teacher^d^	F	4	4–5
3	Preschool care worker^d^	F	34	2
	Preschool care worker^d^	F	12	7–10
	Preschool care worker^d^	F	12	14
	Preschool care worker^d^	F	10	3–4
	Preschool care worker^d^	F	8	“Some”

^a^ Professionals with university degrees in Preschool Education with add-on education for principals.

^b^ Professionals with university degrees in Preschool Education and further education in special education.

^c^ Professionals with university degrees in Preschool Education.

^d^ Professionals with upper secondary education.

For the most part, the participants will be referred to as participants or preschool staff. Sometimes, when it is important for the content, the principals will be referred to as principals and the special education teacher as special education teacher.

### 2.4 Recruitment

In the recruitment, a purposive sample strategy was employed, specifically targeting participants aligned with the study's aim. Knowledge and experience of the intervention program based on the ESDM were required. They should have participated in the intervention program for at least one year and worked with at least two children. In addition, the participants should have attended an education about autism and ESDM strategies. For information about the education, see 2.8 Procedure. To capture an organizational perspective, we included the principals, even if they did not meet children with autism directly. However, they have attended autism education and were involved in the interventions' planning and follow-up. Furthermore, the principals supported the preschool teachers and preschool care workers in their direct work with the children.

To be able to shed light on our research questions from different perspectives, the participants were recruited from several different preschools. Also, education levels and professions, as well as years of experience working with autism and the intervention program, varied among the participants.

Together with the principals, a list was created with the preschool staff who met the inclusion criteria. The participants were chosen with a focus on getting various education levels, professions, and years of experience working with autism and the intervention program. Potential participants were contacted by the first author. If they accepted to participate, their consent was duly sought, accompanied by a thorough review of the study through oral and written informed consent. All participants, but one, accepted involvement in the study.

### 2.5 Data collection

Data were collected through focus group interviews. The interviews followed one of two semi-structured interview guides developed by three of the authors (PL, EC, and GN), who have extensive clinical experience working with young children with autism and working with the ESDM. PL (clinical psychologist/preschool teacher) and GN (pediatrician/child and youth psychiatrist) are members of the multidisciplinary team. In addition, PL and GN are certified ESDM therapists and have developed the intervention program based on ESDM. EC (speech language pathologist) has special knowledge of the qualitative method and in conducting interview studies.

The interview guides have been developed by abstracting the aim of the study, reviewing the literature, and using clinical experience. There were two interview guides, one for staff who worked daily with the children, and one for staff who worked indirectly, i.e., the principals and the special education teacher. However, both interview guides contained open ended questions, for instance, “*Tell me about the work with the intervention program.*”, “*How have you worked with the strategies in everyday life*?” and “*What opportunities and obstacles have you experienced in working with the intervention program?*”. If necessary, the interviewer asked follow-up questions to cover different aspects of the topics.

The last author performed all interviews. The interviewer was unknown to the participants, allowing them to talk more openly about their experiences. Each focus group met on one occasion, and the interviews lasted 60–70 min. They were held in Swedish and were conducted at the local family health center in the study area. The interviews were audio recorded and subsequently transcribed verbatim by PL. Data saturation was continuously discussed between PL and EC and was estimated to have been reached after the third group interview, i.e., the point in the research process where sufficient data has been collected to draw essential conclusions.

### 2.6 Data analysis

Data analysis was carried out by authors PL and EC. Data were organized and structured with the software program NVivo 12 (61). Data was analyzed and interpretated in close collaboration and regular discussions between first and last author by according to content analysis described by Graneheim and Lundman (55). The data analysis started in connection with performing the interviews, where EC, who conducted the interviews, made memos, and discussed those with PL afterwards. PL made the transcriptions, and next step was to read all transcriptions very thoroughly to get an overview of the material as a whole.

Then, a structural analysis continued by sorting the material into meaning units, each representing a single content item. After that, the material was sorted into condensed meaning units, i.e., shortened but with preservation of the content. Some parts of the text material were not related to the aim, and therefore not included within the analysis. For example, the participants discussed how they earlier worked with special groups for children with multiple disabilities. When all material was analyzed and all meaning units detected, the units were labelled with a code (62). PL coded most of the dataset and continuously discussed ambiguous codes and then later subthemes and themes with the last author to ensure methodological rigor and trustworthiness of the results (63, 64). The analysis included a back-and-forth movement through the described steps, with a later decision sometimes altering earlier parts of the analysis. Then similarities and differences between codes and across interviews were reflected upon and discussed, sorting them into themes and subthemes. The interpretation of the themes and subthemes were continuously revised and refined. Quotations have been included within the findings section to illustrate and verify the interpretation and to increase trustworthiness (55). Since the interviews were conducted in Swedish, the quotations were translated into English. In the result, it is displayed from which focus group the quotations are taken, see Table 1. Table 2 provides example of the analytic process from quote to theme.

**Table 2:** Examples from the analytic process, the content analysis—from quote to theme.

Theme	Subtheme	Code	Quote
General key elements	The principal's role—to create prerequisites for the work	Organizational conditions, where the principal is the one who provides the conditions for collaboration.	*”It is up to the principals to give us those conditions. If the principals also prioritize this, then they will prioritize that we can get away. How they choose to organize the activities and how they plan their budget.”*
	The local team as a resource—only a phone call away	The physical proximity, which allows everyone to attend the network meetings.	*“I think it is crucial for us to be able to have the cooperation that we have. I can say that we would not have been able to get away if we had had to go somewhere.”*

### 2.7 Procedure

#### 2.7.1 The intervention program based on the ESDM

In the intervention program, ESDM strategies were used by the parents and preschool staff in the child's natural settings for 24 months. A certified ESDM therapist^3^ coached the parents and preschool staff to use every day routines to create opportunities for interaction and communication, including gaining the child's attention, promoting dyadic engagement and joint activity routines, as well as enhancing verbal and nonverbal communication (16, 23).

Some adjustments were implemented to adapt to the needs of families with multiethnic backgrounds, including simplifying the objectives and learning steps. The therapist orally guided parents through the various steps. If necessary, the therapist visited the child's home and the preschool to implement the objectives and ESDM strategies in everyday life. Also, a simplification of language was necessary, as some of the parents could not read Swedish or were illiterate. Interpreters were used in more than 60% of the appointments. However, fundamental aspects of the ESDM methodology were maintained.

**Figure 1:**
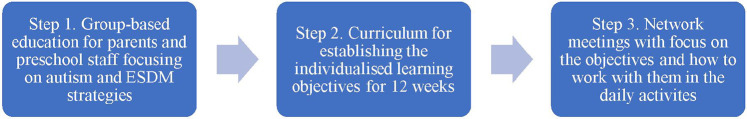
The intervention program based on the ESDM and the different steps (step 1–3).

The intervention program included three steps (Figure 1).

Step 1. Group-based education for parents and preschool staff

The parents and preschool staff were engaged in an education program centered on autism and ESDM strategies. The education comprised information of autism indicators, underlying factors contributing autism, and co-existing conditions. Additionally, it provided a theoretical background on the ESDM and strategies for fostering avenues of communication and interaction within daily routines. Preschool education also included strategies to facilitate interaction between children with autism and their typically developed peers, e.g., working in small groups and encouraging naturally occurring interactions with peers. For the parents, the education was a live presentation and spanned 3 h over two different occasions. During the education, the parents were also allowed to discuss their experiences of having a child with autism with each other. The preschool education was a group-based digital interactive presentation and included a single section of 3.5 h.

Step 2. The ESDM Curriculum Checklist for Young Children with Autism for establishing individual objectives

The ESDM curriculum was used to widely assess the child's skills across various developmental domains (receptive communication, expressive communication, imitation, play, joint attention, social skills, cognition, fine motor, gross motor, behavior, and personal independence) and to establish individualized teaching objectives. The ESDM therapist utilized the curriculum to formulate specific objectives for the individual child. These objectives were also based on the parents' learning profile and identified priorities, i.e., areas deemed particularly important to work on within the home environment (65). Moreover, the preferences and prerequisites of the preschool, including available resources, were considered. At the first network meeting, the therapist presented the objectives for the parents and preschool staff, and the objectives were discussed, e.g., how to work with them at home and in preschool.

Usually, the curriculum was administered every 12 weeks, and new objectives were formulated when old objectives had been fulfilled. An objective was accomplished when the child achieved the task across activities, settings, and adults (about 80% of the opportunities).

Step 3. Network meetings

**Figure 2:**
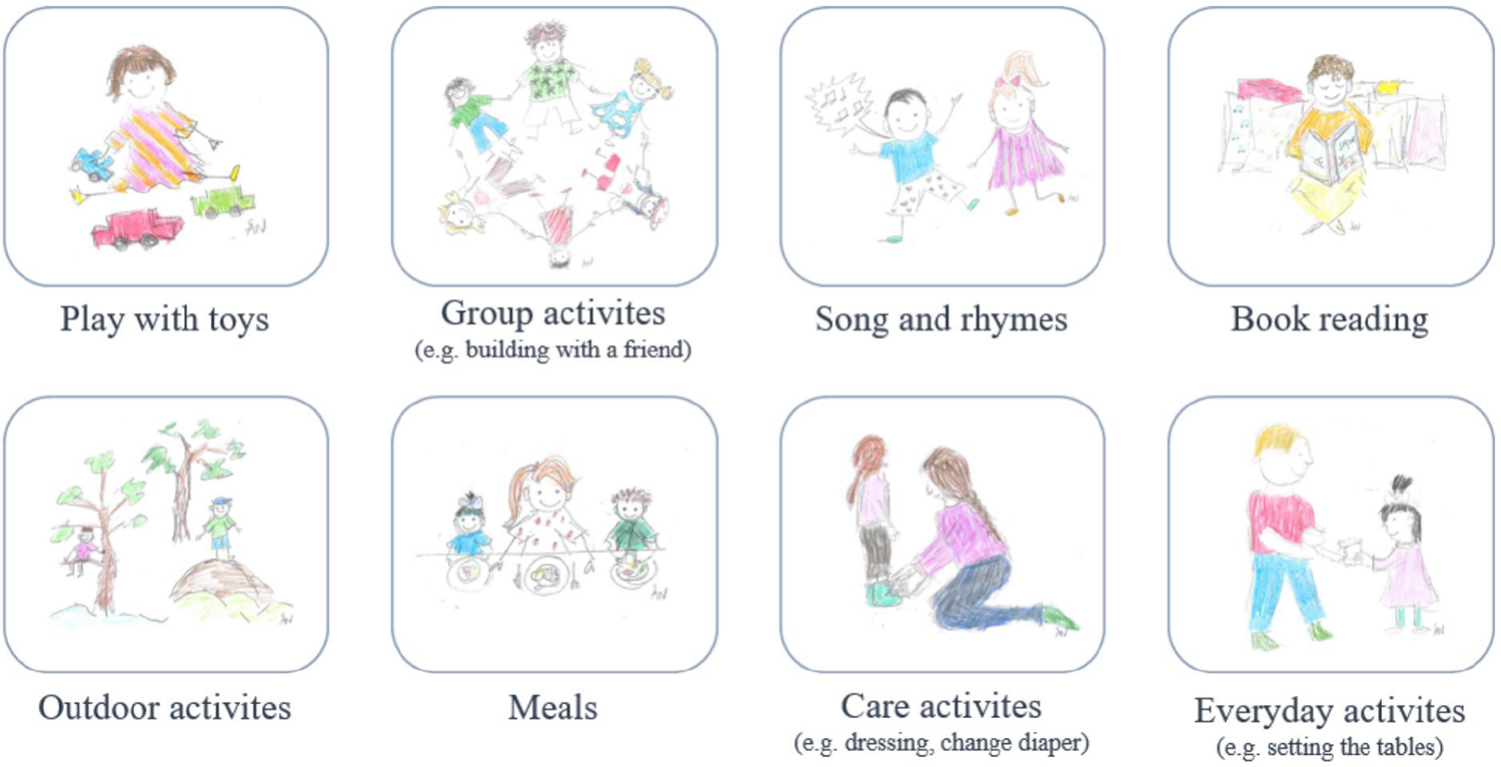
The daily activity categories used in the preschool setting [created by Linnsand, 2019, inspired by Vismara (66), illustrator: Å. Nylander, O. Linnsand].

Every second week, a network of individuals, including the child, the parents, the preschool staff, and the ESDM therapist, convened. While most meetings took place at the clinic, occasionally they occurred at home or preschool. The sessions followed a recurring structure, including a greeting ritual, playtime, song and rhymes, a break for snacks, book reading, and a goodbye ritual. The therapist coached both the parent and preschool staff on implementing the ESDM strategies and achieving the objectives in the child's day-to-day life. The therapist explained and modelled the strategies, and the parents and preschool staff could also practice them and receive feedback. The parents and preschool staff performed the objectives in the child's daily activities between the network meetings. The daily activities at home included bathing, reading books, dressing/nappy changing, household tasks, meals, outdoor activities, sensory social routines, and playing with toys. Figure 2 illustrates the daily activities in the preschool.

During the network meetings, the preschool staff got advice on facilitating learning through peer interactions and social participation. For example, when peers did something interesting, e.g., collapsing a tower, how could the preschool staff encourage the child to pay attention to and imitate the play action? Likewise, when the child with autism did something fascinating, how could the preschool staff encourage peers to engage in the play? Strategies from Vivanti et al. (67) were used.

## 3 Results

**Figure 3:**
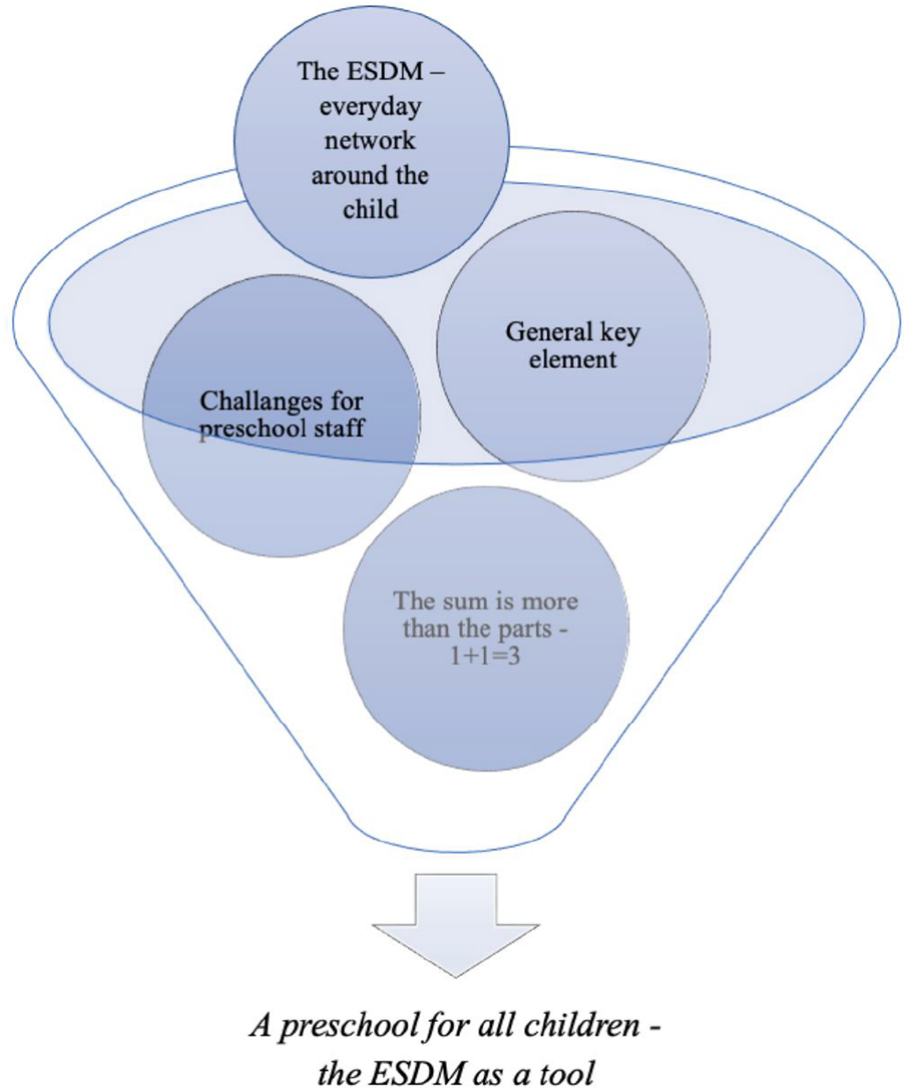
Four main themes emerged, which constituted the essence of the participants’ experiences.

According to the preschool staff' narratives, the analysis revealed four main themes and 10 corresponding subthemes pertinent to the objectives. The overarching theme pervading the main themes and subthemes was “*A preschool for all children—the ESDM as a tool*”, Figure 3. The initial facet of this theme encapsulated the participants' experiences that the intervention program significantly enhanced the educational environment for children with autism*.* i.e., “*A Preschool for all children*”. Furthermore, they experienced that the ESDM provided tools in establishing a preschool environment where children, including those with autism, were exposed to stimulating and challenging learning experiences.

The first main theme that emerged from the analysis, “*The ESDM—everyday network around the child*”, comprised two subthemes. The second main theme, “*General* *key element*”, included three subthemes. “*Challenges for preschool staff*” was the third subtheme, comprised two subthemes. The last one, “*The sum is more than the parts—1 + 1 = 3*”, included three subthemes. Table 3 listed the main themes and subthemes.

**Table 3:** Overview of the overarching theme, main themes, and subthemes (*n *= 15).

Overarching theme	A preschool for all children—the ESDM as a tool
Main themes	Subthemes
The ESDM –everyday network around the child	Shared objectives and collaboration Easy to adapt to the preschool curriculum and in daily activities
General key elements	Principal's role—to create prerequisites for the collaboration Local team as a resource—only a phone call away Knowledge of each other's missions and responsibility
Challenges for preschool staff	Communication with parents about the child's development—the role of knowledge mediator Not just one child with autism—to provide education for all children
The sum is more than the parts – 1 + 1 = 3	Valuable for several—for the child with autism, the fellow peers in preschool, and the parents Improved collaboration—increased communication and consensus around the child's needs New knowledge creates security

### 3.1 The ESDM—everyday network around the child

The first main team centred on the ESDM as the intervention model and comprised two subthemes. These subthemes encapsulated crucial facets of the ESDM.

#### 3.1.1 No 1. Shared objectives and collaboration

The participants emphasized the significance of shared objective-setting and regular network meetings as integral components of the ESDM. The shared goal setting was central in fostering enhanced collaboration and communication with the parents. The participants expressed that the objectives contributed to creating a shared platform where everyone worked similarly. Also, the shared goals eased the transition between preschool and home when the caregivers used similar strategies in their daily routines. In addition, the participants experienced that collaboration contributed to increased opportunities for the child to generalize learned skills across different adults and settings. “*The exercises we do, they do exactly at home*.” (Focus group 2).

Another aspect of the shared objectives and the network meetings was the opportunity to learn from each other. During the network meetings, the ESDM therapist provided the preschool staff and parents with opportunities to employ strategies and interact with the child. Sometimes, the parents modelled how they used the strategies, and the preschool staff could employ the same strategies in preschool and vice versa. Also, the meetings provided opportunities to discuss behavioural challenges. “*You can ask for help from the preschool as well. How does my child do when it comes to being outside? How do you handle the food situation?*” (Focus group 2).

For the child with autism, the shared goals contributed to more predictable routines in the preschool setting. All preschool staff were familiar with the objectives and were, therefore, able to implement them in daily activities. “*Everyone has the same mindset throughout the day.*” (Focus group 1).

Furthermore, the shared objectives contributed to preschool staff being able to evaluate their efforts. “*The goals that you said, that we set together here at Hjällbo collaboration, also help you to kind of focus and see the progress*.” (Focus group 3)*.* In the past, they had found it difficult to see progress in the child's development. When they noticed the improvements, they experienced reduced stress and increased confidence in their professional role. “*I have got a more relaxed attitude towards working with children with special needs*.” (Focus group 3).

#### 3.1.2 No 2. Easy to adapt to the preschool curriculum and in daily activities

The participants experienced an alignment between the ESDM and the preschool curriculum. “I*f you had to find objectives for the curriculum /…/ this would not be a problem for all exercises.*” (Focus group 2)*.* They emphasized communication, interaction and play as central tenets within the ESDM and found those areas easy to attach to the preschool curriculum. “*Because we have communication, for example language is a big part of the curriculum*” (Focus group 2). Also, they felt familiar with the ESDM way of looking at development and learning, i.e., the focus on the child's interests and motivation to create joyful learning experiences. *“It comes in there as well. The pleasure of learning*.” (Focus group 2).

The participants experienced that ESDM strategies could be easily integrated into the preschool's daily activities. They used the strategies in various activities, such as feeding, outdoor activities and playing with toys. A participant explained how they use different routine situations as learning opportunities. “*Teaching happens during routine situations as well. Dressing, playing and teaching are treatment*”. (Focus group 1).

The ESDM strategies contributed to creating an education milieu where the children with autism became more involved in the preschool's daily activities. *“The exercises, we usually do them with everyone. Sometimes you don’t have time to just sit down, but they are still exercises that all children enjoy*.” (Focus group 2)*.* Doing the exercises in the group also had other benefits, such as dealing with the lack of time.

### 3.2 General key elements

The second main theme, *General key elements,* comprised three subthemes, focused on fundamental prerequisites crucial to the intervention program, including the principal's role and the locally located healthcare team (Table 3).

#### 3.2.1 No 1. Principal's role—to create prerequisites for the collaboration

The participants described the crucial role of the principal's attitude and involvement in the intervention program. The principals’ attitude to the therapist's work and cooperation with the team was a crucial aspect. *“Our role as principals is very important because we are the ones who /…/ set the status that this is important*.” (Focus group 1).

Also, it was deemed important that the principal took concrete steps to facilitate the preschool staff's involvement, for instance, in the network meetings and the education about autism and ESDM strategies. “*It's up to the principals to give us those conditions. /…/ That you can bring in someone to cover.*” (Focus group 2). The participants also underscored the principal's pivotal role in creating conditions for working with the child in daily activities. For example, work in smaller groups to create learning opportunities more easily for children with autism. In that effort, the participants described various creative solutions. “*She never slept, so we made sure that a teacher had that time with her, and it was that time that was very valuable*.” (Focus group 3).

#### 3.2.2 No 2. Local team as a resource—only a phone call away

The second subtheme captured aspects of the teams' local location and the therapists' availability for support. The participants highlighted the locally located team as one of the most essential prerequisites for collaboration. In the previous cooperation with healthcare, they had travelled approximately one hour by bus to attend the network meetings. “*This is crucial for us to be able to have that cooperation. I can say that we would not have been able to get away if we had had to go somewhere*.” (Focus group 3)*.* Also, they emphasized that the local located team contributed to the parents’ confidence. “*Here you feel at home in some way /…/ You have more confidence*.” (Focus group 3).

Another aspect of the local location was the therapist's possibility to visit the preschool. Then, the therapist could guide the preschool staff on implementing the ESDM strategies and objectives in the preschool's routines. In addition, the therapist's attendance contributed to the shared goals becoming more adapted and feasible in the preschool context. “*They also see what kind of environment we have at the preschool, what kind of group composition we have. What our conditions are like. Because otherwise it's easy to just write a program, do this, but can we do it concretely?*” (Focus group 2).

Another essential part of the collaboration was the therapists' availability for support. The preschool staff described different types of support, for instance, supervision from the therapists. “*It can happen that I don’t know what to do, in which case I’ve a great contact with Petra who can come in and help us with what to do*.” (Focus group 2)*.* Additionally, they described more emotional support, e.g., having someone to share difficulties with and, through discussions with the team, finding new ways to move forward. Sometimes, the participants found communication with the parents challenging, i.e., how to talk about the child's difficulties. Also, in these cases, they found the therapist supportive. “*How to explain in a way that doesn’t hurt but is still realistic. It's nice not to have to do that whole thing yourself*.” (Focus group 3).

#### 3.2.3 No 3. Knowledge of each other's missions and responsibility

The preschool staff had previously cooperated with healthcare professionals in interventions based on the Intensive Behaviour Treatment (68). In these collaborations, the participants experienced that the healthcare professionals wanted them to carry out treatment and decided the framework for collaboration. They described it as the preschool becoming “*as an extension of the healthcare system*.” (Focus group 1). The participants expressed that they lost their mission, i.e., education. Therefore, they highlighted that knowledge of each other's missions, as well as confidence in one's own mission, were crucial in this collaborative. “*Everyone is confident in their own assignments, but they know the other's, so they can complement each other*.” (Focus group 1)*.* For example, individual treatment vs. group teaching was a possible contradiction between the different missions. The participants found it necessary that healthcare professionals had knowledge of the preschool's methods with a focus on group learning and could adapt the ESDM tasks to the group setting.

In earlier cooperation, the preschool staff experienced different terminology used in preschool and the healthcare system. For instance, in healthcare, the professionals used words such as “treatment” and “training”, and, in preschool, “education” and “learning”. Therefore, medical terminology needed to be translated into educational language. In this program, they experienced the transition facilitated the healthcare staff's knowledge of the preschool curriculum and daily routines. “*In a way, it's like translating medical treatment or training into the preschool's educational activities. /…/ Then it may not be needed so much because Hjällbo collaboration employees are so well acquainted with the preschool's activities*.” (Focus group 1)*.* Also, the special education teacher was highlighted as an essential person in the transition process.

### 3.3 Challenges for preschool staff

As shown in Table 3, the third main theme centred around two facets of challenges in implementing the intervention program. The first aspect revolved around communication with the parents, while the subsequent aspect focused on the high prevalence of children affected with autism in the area.

#### 3.3.1 No 1. Communication with parents about the child's development—the role of knowledge mediator

The participants described communication with the parents as challenging, i.e., how to talk about the child's difficulties. “*You don’t always know how to express yourself either /…/ Can I talk about the word difficulty with this parent or can I not*.” (Focus group 2)*.* Sometimes, alerting the parents that their child had a delayed development was challenging. In that context, the participants described different cultural aspects. For example, they talked about how some cultures see abnormalities as shameful. The participants experienced that the parent's shame of having a child with difficulties sometimes prevented them talking about the delayed development and seeking support.

In addition, several parents had limited knowledge about autism and sometimes had never heard the word autism. In almost daily meetings with the parents, the participants became mediators of knowledge. “*I go to my home country, and my child will be fine when we come back in a few months. But we are trying to say that this is not really how it works and autism is something that you have so to speak*.” (Focus group 2).

#### 3.3.2 No 2. Not just one child with autism—to provide education for all the children

In their preschools, the participants noticed that a considerable number of children had autism and/or other NDDs.

*Participants 1: “One child is fine.”*
*Participants 2: “Two children are also fine.”*
*Participants 1: “I counted that we had seven children last semester.”* (Focus group 2).

They discussed the complexity of providing education that catered to the diverse needs of both children with and those without disabilities. For example, they experienced they needed more time to do the trainings with the children affected by autism.

### 3.4 The sum is more than the parts—1 + 1 = 3

The fourth theme that emerged from the analysis, “*The sum is more than the parts—1 + 1 = 3*”, was related to the effect of the intervention program and comprised three subthemes (Table 3).

#### 3.4.1 No 1. Valuable for several—for the child with autism, the fellow peers in preschool, and the parents

The preschool staff described that the intervention program yielded benefits not only for the child with autism but also enduring positive effects on their peers, their parents, as well as for themselves. They experienced that the ESDM positively impacted the child with autism, including improved communication. The progress was linked to the shared goals, for example, a child who had started to use pointing or handled transitions more easily. Some improvements were defined in relation to the other children. “*They can just roll the ball. That's a big improvement. That they can play together*.” (Focus group 3). Also, the intervention program seemed to be advantageous for all children in the preschool, as most children spoke a home language other than Swedish and needed to develop their vocabulary and communication abilities.

The participants experienced that the parents were satisfied and benefited from participating in the intervention program. The therapist coached the parents to implemented ESDM strategies in daily life, establishing functioning routines, and reducing behaviour problems. The therapist's possibility to make home visits was highlighted as particularly valuable, for example, guiding the parents in mealtime situations. Emotional support for the parents was emphasized as essential and parental emotions such as grief, sadness, shame, relief, and confidence were described. “*I’ve noticed that it's a relief and a little sadness too, but security*.” (Focus group 2).

#### 3.4.2 No 2. Improved collaboration—increased communication and consensus around the child's needs

The second subtheme underscored the collaboration between the parents, the participants, and the healthcare professionals. The participants expressed that the network meetings contributed to enhanced collaboration and improved communication with the parents. “*Then we end up in those dialogues, where we don’t understand each other. It's not just us and when you come here and can talk and open up everything*.” (Focus group 3). The collaboration brought the child's different arenas together, which led to a consensus on the child's needs and increased involvement in the interventions. “*One person does not own the issue and the others are participants, but we own the issue together in the best interests of the child*.” (Focus group 1).

#### 3.4.3 No 3. New knowledge creates security

The most prominent content of this subtheme was the increased knowledge about autism and ESDM strategies. “*It is competence development /…/ We have broadened the base*.” (Focus group 1). The participants gained new knowledge when participating in the education program as well as in the network meetings. They talked about how they received new strategies to fostering avenues of communication and interaction within the daily activities, but also about tools to understand and solve challenges that may arise in the child's everyday life in preschool. A participant expressed that she has become more aware of the small details in the interaction, for example, catching the child's attention by holding the object up to the eyes.

The increased knowledge and new tools have contributed to increased confidence. “*There is also a different kind of calm among the teachers. You don’t start to worry /…/ You know that you can solve it*.” (Focus group 1). Also, the participants expressed that advanced knowledge and new tools improved the work environment, thus achieving a sense of control and security. “*Training and teaching will be improved, as well as the working environment for teachers*.” (Focus group 1).

The heightened knowledge facilitated the early recognition of signs indicative of autism. Also, it contributed to the fact that the preschool staff could start interventions in the child's everyday life before the full assessment had been completed. “*Working with new children is not so difficult. You know what to do*.” (Focus group 3).

## 4 Discussion

The present study aimed to describe an intervention program based on the ESDM in a resource-strained multiethnic immigrant context, while also seeking to encapsulate the perspectives and experiences of preschool staff engaged in implementing the program. The overarching theme was *A preschool for all children—the ESDM as a tool*. This theme captured the participants’ experiences that the intervention program significantly improved the educational environment for children with autism. The ESDM provided tools for establishing an educational setting where children, including those with autism, obtained stimulating and challenging learning experiences. It aligns with the preschool curriculum, which emphasizes the inclusion of children with special needs in regular preschools (38). In that effort, the preschool staff expressed both facilitators and barriers.

**Figure 4:**
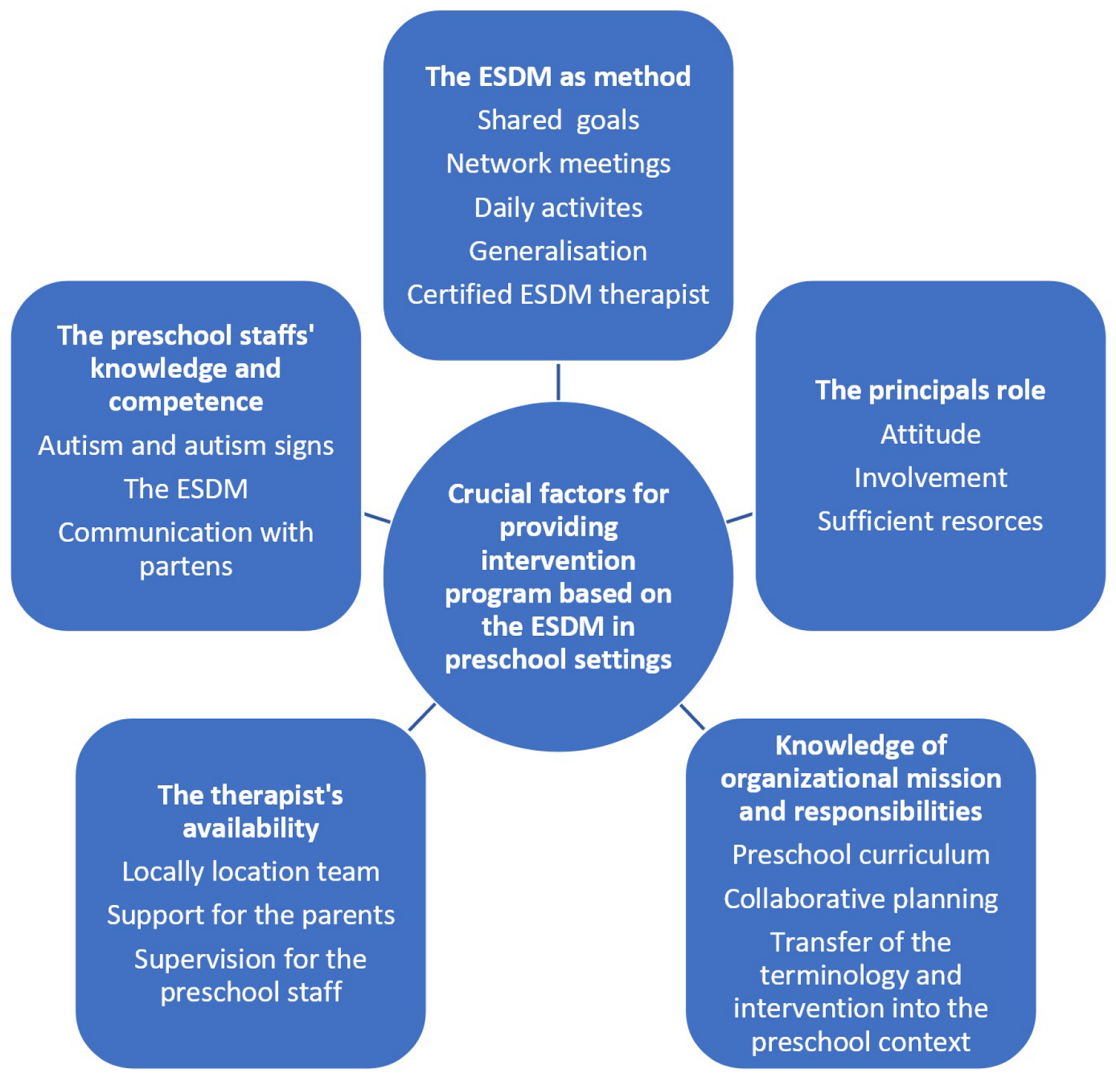
A model of crucial factors for providing intervention program based on the ESDM in preschool settings.

The analysis revealed that there were some fundamental prerequisites for the intervention program, including the principal's role and the team's location. In Figure 4, we have formed a theoretical model to illustrate crucial factors for delivering the intervention program based on the ESDM in the preschool context. In this model, we have incorporated knowledge and competence as critical elements. We suggest knowledge about autism, in general, is essential in providing a high-quality intervention, but also knowledge and competence in the intervention model, i.e., the ESDM. This is in line with earlier research (69, 70). In a Swedish study by Westman Andersson et al. (71), parents highlighted the importance of competence and knowledge about autism among preschool staff, including the principal.

The preschool staff highlighted the seamless alignment between the ESDM and the preschool curriculum, for instance, the consistency between the perspective of the child's development and learning. Play-based learning and the cultivation of relationships with an emphasizes on language and communication are crucial tenets within both the preschool curriculum as well as in the ESDM (16, 38). Also, in other studies, the ESDM has been considered compatible with preschool routines and the ESDM strategies fitted naturally into preschool teaching practice (33, 34). In that sense the model could be considered as having a high social validity (72, 73). In Sweden, the most common intervention program for young children with autism is the EIBI, while challenges in implementation in regular preschools have been reported (35, 36, 69). For instance, the preschool staff has described obstacles in collaboration with healthcare services (36) and some of the training principles of the EIBI may be controversial within the preschool context (74). In our study, the participants experienced that the ESDM strategies could be easily integrated into the preschool daily activities. This is likely due to several factors, but we will discuss two different aspects.

Firstly, we propose that the naturalistic approach, i.e., creating learning opportunities in the child's everyday life, might be essential. Also, the techniques in the ESDM are appealing for preschool staff, including natural reinforcement, child-initiated teaching episodes, and modelling (14). Secondly, the shared objective-setting was essential and contributed to the ESDM being perceived as easily integrated into the preschool setting. The fact, that the objectives were designed based on the identified priorities and characteristics of both parents and preschool staff simplified the implementation (65).

Vivanti et al. (75) have identified factors, including infrastructure and resources, staff motivation/commitment, and organizational leadership as influencing intervention implementation in preschool settings. In the present study, contextual factors were addressed to facilitate the implementation of the intervention program, including the availability of the healthcare team and the principal's role. The principals had a pivotal role, consistent with previous research from Sweden (69). The principal “set the agenda” for the program and facilitated the implementation, for instance, to enable the participants' involvement in network meetings and the education program. Another contextual factor was the healthcare team's availability, both the local location and the therapist's availability for support. The preschool staff emphasized the significance of the multidisciplinary team's local attachment as a prerequisite for effective collaboration. In addition, they underscored the importance of ESDM therapist's availability for support. The therapist coached the preschool staff and the parents, applying the child's individualized objectives in everyday activities. During the network meetings, the therapist provided opportunities for both preschool staff and parents to practice strategies with the child, offering constructive feedback and support. Earlier research has shown that coaching may be effective, when it takes place within the educators' context, is individualized, provides practice new skills, and sets self-directed goals (76).

The therapist's expertise and understanding of the preschool curriculum and daily life were instrumental in cultivating collaboration and implementing the interventions in the preschool setting. In previous collaborations, the preschool staff experienced that they sometimes “lost their way”. There was a conflict between the preschool's educational approach and the healthcare's medical perspective. The differences became visible, for example, in medical and educational terminology, such as education vs. treatment. Another example was the healthcare's focus on the patient and the preschool's focus on the group, where most preschool activities occur in groups rather than individually. In the study by Roll-Pettersson et al. (69), the educators expressed that the different missions and values could lead to tensions, whereby the preschool's behavioural special educator could create a bridge between the organizations. The participants highlighted the preschool's special pedagogue as a possible bridge between the preschool and healthcare. In addition, the bridging appeared facilitated by the therapist's preschool expertise. For instance, the medical terminology needed to be translated into the educational language, and the tasks had to be adapted to group settings.

Two challenges were identified in implementing the intervention program, including communication with the parents, while the second aspect concentrated on the high prevalence of children with autism in the area. The participants experienced it was challenging to talk to the parents about the child's deviant development, and different parental cultural backgrounds made it more complex. In a review, Kärtner et al. (77) have found that sociocultural expectations and family experiences have a decisive role in whether a social communication behaviour is considered “typical” or “concerning”. Also, stigmatization can contribute to parents' resistance to seeing the deviant development and reduce the willingness to seek support (78, 79). The preschool staff experienced the parent's shame of having a child with difficulties that sometimes prevented them from talking about the deviant development and seeking support. Research has shown cultural differences in the awareness and knowledge of autism and autism signs (41, 80–82). Donohue et al. (82) have found racial differences in the concerns that parents reported to healthcare providers, such as concerns about autism, social interaction, and repetitive behaviour. Further, the preschool staff noticed that some parents had limited or no experience and knowledge about autism. For instance, Selman et al. (83) found that Somali parents lacked awareness of autism as a condition, and few words in the Somali vocabulary were related to autism. In the present study, the preschool staff had a pivotal role as knowledge mediator.

The subsequent challenge might partly be linked to the high prevalence of autism in the study area (58). Several studies have suggested that the risk of receiving an autism diagnosis is higher among children of immigrants than other children, especially for low functioning autism (41, 42, 84–87). Probably, other factors also impact the challenge of providing education that catered to the diverse needs of both children with and without disabilities. In a report from the Swedish National Agency for Education, staff density, the number of children in the groups, the preschool staff's competence and education, as well as the local's design were highlighted as factors that influence the preschool quality (88). Pramling Samuelsson et al. (89) have demonstrated that with fewer children in the group, preschool teachers experienced that they, to a greater extent, can work in line with the preschool curriculum intentions.

The participants highlighted that the intervention program generated benefits not only for the children with autism but also might have positive effects on their peers in the preschool, the parents, and the preschool staff. The participants perceived that the ESDM positively impacted children with autism, such as improved communication and interaction. Several studies have demonstrated the efficacy of preschool based ESDM, including improvements in core developmental domains and decreased maladaptive behaviour (27–31). Additionally, the preschool staff experienced that the ESDM proved advantageous for other children in the preschool. Given that most of them spoke a home language other than Swedish, the ESDM offered them valuable chances to develop their vocabulary and communication. Also, the network contributed to enhanced collaboration and improved communication with the parents. The child's different worlds were brought together, which contributed to increased involvement for both parents and them.

The preschool staff's knowledge is essential in providing a qualitative educational environment, where children with autism could engage in the preschool activities. A report from the Swedish School Inspectorate showed only one third of the preschools were considered to be providing sufficient support for children with special educational needs (90). Increased knowledge about neurodevelopmental disorders among educators has been highlighted as an essential measure (91). The preschool staff in the present study received education on various facets, including autism, underlying factors contributing to it, the theoretical framework of the ESDM, and strategies to enhance opportunities for communication and interaction. An inclusive environment can provide children with autism opportunities to observe appropriate social communicative behaviours and to practice these behaviours with their typically developing peers. But to become inclusive, we suggest that the preschool staff's knowledge and an awareness approach are necessary. Otherwise, it is a high risk that the children with autism are included but not integrated (92).

Finally, the increased competence also facilitated the early recognition of signs indicative of autism. The preschool staff used ESDM strategies even before contact with the healthcare system was established. This is an essential finding based on research on critical developmental periods of brain growth and the importance of early intervention (7–10). In a previous study from our research group, we suggested that the preschool setting may be an essential part, together with Child Health Center, in the early identification of autism indicators (93). Children from low resource settings are typically diagnosed with autism later than their general peers (41, 94, 95). In addition, opportunities for autism interventions, both in care and preschool, have been lacking in immigrant populations (41). In earlier studies, we have underscored the imperative to develop new multidisciplinary models in healthcare to increase accessibility to services for children with autism in immigrant communities (58, 93).

### 4.1 Methodological considerations

As the current study uses a qualitative methodology, the findings reflect the participants' subjective, individual experiences. The methodological aspects of trustworthiness were considered in terms of credibility, dependability, confirmability, and transferability (96). By recruiting a divergent interview group, aspects of credibility have been considered. A larger sample of participants might have contributed to more aspects of experiences. However, interviews were conducted until the point of saturation, which in itself supports the credibility of the findings. Also, the authors' different backgrounds and perspectives were important in the analysis, which could be approached from several angles and perspectives. Also, the interviewer was not personally involved in the intervention program to handle the truthfulness.

Dependability was strengthened by using a semi-structured question guide for focus group interviews to ensure that the aim of the study remained in focus. Discussions were held continuously during analysis to enhance reflexivity, and sometimes consensus decisions were taken. While selecting meaningful units, the work started in collaboration between the first and last author. The first author continued choosing units and, several times, checked with the last author (64). Confirmability has been considered using the presentation of quotes from the interviews to illustrate the connection between the interview data and the analysis. Finally, transferability was considered regarding the importance of describing the included participants (97).

## 5 Conclusion

To summarize, the preschool staff's experiences indicated that implementing the low intensity intervention program based on ESDM enriched the learning experiences of children with autism. Based on the participants' experiences the ESDM strategies seem feasible for integration into preschool daily routines and aligned with its curriculum. However, more research is necessary to evaluate the usefulness and efficacy of this intervention program.

Several critical factors based on the preschool staff's experiences are essential for implementing interventions for children with autism in a preschool setting. Firstly, contextual prerequisites are required, for instance, organization support. Secondly, close collaboration with healthcare and good competence of the preschool staff are instrumental to succeed in implementation. Third, an evidence-based intervention method should be used, but adapted to the preschool context. The analysis underscored the pivotal role of the ESDM itself as a key factor in the intervention program's implementation within the preschool setting.

## Data Availability

The datasets presented in this article are not readily available because data is not anonymized. Therefore, access to data can be granted after an approved application to The Ethical board Sweden and other internal legal procedures authorize external access to the data. Requests to access the datasets should be directed to petra.linnsand@vgregion.se.

## Footnotes

^1^Individuals born abroad or born in Sweden with both parents born abroad.

^2^Children born abroad or in Sweden with both parents born abroad.

^3^In the intervention program, there were three therapists, two of whom were certified ESDM therapists. The third therapist has worked with interventions for young children with autism for 26 years and has experience with both the ESDM and methods, according to the EIBI. However, in the intervention program, she worked with interventions according to the ESDM. She has several courses in the ESDM. Furthermore, she also received supervision from the certified ESDM therapists.
